# A novel route for identifying starch diagenetic products in the archaeological record

**DOI:** 10.1371/journal.pone.0258779

**Published:** 2021-11-18

**Authors:** Thomas Oldenburg, Melisa Brown, Jamie Inwood, Jagoš Radović, Ryan Snowdon, Steve Larter, Julio Mercader

**Affiliations:** 1 Department of Geoscience, University of Calgary, Calgary, Alberta, Canada; 2 Department of Anthropology and Archaeology, University of Calgary, Calgary, Alberta, Canada; 3 Department of Archaeology, Max Planck Institute for the Science of Human History, Jena, Germany; 4 Institut Català de Paleoecologia Humana i Evolució Social, Tarragona, Spain; Fisheries and Oceans Canada, CANADA

## Abstract

This work introduces a novel analytical chemistry method potentially applicable to the study of archaeological starch residues. The investigation involved the laboratory synthesis of model Maillard reaction mixtures and their analysis through Fourier-Transform Ion Cyclotron Resonance Mass Spectrometry (FTICR-MS). Thus, starch from sixteen plant species were matured while reacting it with the amino acid glycine. The FTICR-MS analysis revealed > 5,300 molecular compounds, with numerous unique heteroatom rich compound classes, ranging from 20 (*Zea mays*) to 50 (*Sorghum bicolor*). These classes were investigated as repositories of chemical structure retaining source and process-specific character, linked back to botanical provenance. We discussed the Maillard reaction products thus generated, a possible pathway for the preservation of degraded starch, while also assessing diagenetic recalcitrance and adsorption potential to mineral surfaces. In some cases, hydrothermal experimentation on starches without glycine reveals that the chemical complexity of the starch itself is sufficient to produce some Maillard reaction products. The article concludes that FTICR-MS offers a new analytical window to characterize starchy residue and its diagenetic products, and is able to recognize taxonomic signals with the potential to persist in fossil contexts.

## Introduction

Paleoanthropologists have hypothesized that hominin diet and society underwent major transformations upon reliance on starchy food, with primitive and archaic hominins consuming simple carbohydrates from fruits [1], rhizomes, tubers, nuts, seeds, legumes, and piths [2–4]. Starch consumption presumably drove the emergence of cooking, cohesive social systems ruled by sharing [5], and in turn, the appearance of derived body traits, as well as new technologies such as nut cracking [6] and grinding stones [7–11]. However, these evolutionary assumptions on the origins and implications of human starch consumption are speculative, given the paucity of direct evidence; for instance, fossilized or charred starchy macroplant remains [12–15].

In the near absence of macrofossils, archaeologists have come to rely on microscopic starch granules to study ancient starch use [e.g. 16, 17]. Unlike true fossils, however, the starch granules from archaeological accounts behave optically like fresh granules, in native state, seemingly impervious to (molecular) degradation. Yet, from a chemical perspective, the idea that highly reactive carbohydrates could bypass diagenesis to stay intact over thousands of years does not reconcile with bio-geo-chemical tenets relating to structural preservation [18] or carbohydrate biodegradation in soils and surfaces [19]. Furthermore, adsorbed biofilms [20], as coatings [21], result in the binding of diagenetically modified organics to mineral surfaces [22]. In contrast, however, dozens of articles [cited in 17] report native granules found in starch slides; many directly pipetted off artifact surfaces, some from partially dissolved dental calculus, while others are dislodged by acoustic cavitation through water. Importantly, after three decades of archaeological inquiry [23–34], whether starch granules thus retrieved are ancient, or just exogenous, simply remains unproven in the absence of direct evidence that these are authentic starch ‘microfossils’.

In this paper, we focus on diagenetic molecular fingerprints from carbohydrates of interest to archaeology as an independent alternative / complementary pathway to study ancient starch. The structural characterization of heterogeneous, heteroatom-rich compounds, however, would be impossible to accomplish by standard chromatographic and mass spectroscopic techniques because of the wide range of polarity, functional group distributions, molecular weight, and volatility demonstrated by the products from these reactions. In years past, the application of analytical chemistry to study biomolecules has taken on a diversity of perspectives [35–37], with an emphasis on organic residues from adhesives [38, 39], beverages [40], fats [41, 42], and proteins [43, 44]. The systematic application of High Resolution Mass Spectrometry (HR-MS) to the processing and interpretation of archaeological materials is in fact very recent; restricted in scope to ancient lipids from ceramic vessels [45, 46], and utilizing time-of-flight MS [47]. We use ultra-high-resolution Fourier-Transform Ion Cyclotron Resonance Mass Spectrometry (FT-ICR-MS) to analyze compound classes and the species found in complex mixtures of Maillard reaction products (MRP) generated upon non-enzymatic alteration of starch. The starch polymer is conceived as the reactive chemical precursor of complex compound mixtures that result from a variety of handling, culinary, and diagenetic processes; including the Maillard reaction. This occurs during non-enzymatic alteration of carbohydrate and protein, yielding nitrogenous polymers as Maillard reaction products (MRPs) [48–53]. With amino acids and carbohydrates as the feedstock, melanoidins, as end products from the Maillard reaction, bind a variety of functionalized species such as lipids, while on thermal alteration they convert to high molecular weight kerogenous materials [49]. This makes some melanoidin compounds less accessible to microbial degradation and diagenetically stable, allowing characterization within accepted taphonomic paradigms [54, 55].

## Materials and methods

### Sample preparation and characterization

We study modern starch samples from a reference collection to establish a baseline for archaeological chemistry, guiding classification and identification of plant residue from ancient materials by comparison. The focus is on ethnobotanical taxa for food and medicine, encompassing starch granules of diverse crystallinity, morphometry, and for the most part well-understood chemistry (Table 1). As early humans utilized unmodified starches, we also emphasized non-purified starch, yet we included three purified specimens from common grains (*Zea mays*, *Triticum aestivum*) and tubers (*Solanum tuberosum*) for contrast; in which case starch was industrially isolated by steeping in water and several cycles of sieving and centrifuging [56] that remove fiber and protein [57]. All samples came from an established botanical collection at the University of Calgary (Earth Sciences 833). The selected herbarium specimens were cleaned mechanically with sterilized dental brushes and air. The sample was then open with a dissecting tool. From the central portion of the starch-storing tissue, we extracted 1 gram by scraping with a sterilized scalpel. Powdering followed in a previously autoclaved mortar with a pestle by very light grinding in a circular motion and without applying any pressure.

**Table 1:** Taxonomy and characterization of starch samples.

Part	Family	Taxon	Carbon%	Hydrogen%	Nitrogen%	Sulfur%
U.S.O.*	Araceae	*Colocasia esculenta*	35.59	6.43	1.06	0.00
U.S.O.*	Dioscoreaceae	*Dioscorea esculenta*	41.54	6.51	0.76	0.07
U.S.O.*	Dioscoreaceae	*Dioscorea schimperiana*	40.30	6.98	0.34	0.04
U.S.O.*	Cyperaceae	*Cyperus rotundus*	43.71	6.56	0.30	0.08
U.S.O.*	Musaceae	*Ensete ventricosum*	39.58	6.46	0.25	0.10
U.S.O.*	Euphorbiaceae	*Euphorbia quadrangularis*	42.19	6.24	0.34	0.04
U.S.O.*	Vitaceae	*Cyphostemma serpens*	36.38	5.58	0.30	0.00
U.S.O.*	Convolvulaceae	*Ipomoea longituba*	37.52	6.09	0.55	0.61
U.S.O.*	Solanaceae	*Solanum tuberosum*	41.21	6.58	0.45	0.00
Legume	Fabaceae	*Lablab purpureus*	42.36	6.67	8.06	0.50
Legume	Fabaceae	*Mucuna pruriens*	45.13	6.91	4.21	0.19
Legume	Fabaceae	*Senna didymobotrya*	46.37	7.45	10.22	0.71
Grain	Poaceae	*Sorghum bicolor*	41.83	6.51	0.79	0.00
Grain	Poaceae	*Triticum aestivum*	44.08	6.73	3.01	0.15
Grain	Poaceae	*Zea mays*	42.06	6.65	0.00	0.00
Grain	Euphorbiaceae	*Erythrococca bongensis*	54.58	8.97	3.78	0.09

(*Underground Storage Organ).

From a taphonomic / diagenetic point of view, it has been established that biomolecules are not stable on an archaeological time scale, as they transform chemically in short periods [58]. Therefore, controlled laboratory experimentation looking for analogue reactions in deep time justifiably keep temperature and pressure relatively elevated [59] to mimic the chemical changes that would occur during burial and diagenesis over thousands of years. As no degradation products have been claimed for glycine during the Maillard reaction [52, 53], we used glycine (C_2_H_5_NO_2_) in subordinate concentration to ensure that the amino acid itself would not substantially complicate reaction products and to help develop a more prominent carbohydrate related signal in the resulting MRP species. For controls, we experimented on some starch species with and without glycine. And while glycine alone did not produce compounds amenable to FTICR-MS, starch only assays revealed that the chemical complexity of the polymer and its impurities are sufficient to generate some MRPs. However, the hydrothermal reaction products observed do include a range of chemical species that go beyond MRPs, although, for simplicity, all of them are referred to as MRPs throughout the text.

As the starch nitrogenous/sulfur impurities vary among taxa, we normalized all experiments by adding glycine. The ratio of glycine to starch was 0.75 / 1 by weight in a test tube (10 ml). We added reverse osmosis deionized water (RODI: 7 ml) and tested pH (~ 7). This mixture was sealed in a pressure vessel, in triplicate; one vessel kept at room temperature (25°C), one heated at 70°C, and another heated at 150°C for 24 h. Each vessel, post-treatment, was cooled and centrifuged (3000 r.p.m.), collecting an aliquot (300 μl) from the aqueous phase, which received ammonium hydroxide (NH_4_OH: 20 μl) as a pH dopant right prior to analysis. The starch polymer is concurrent with small and trace amounts of proteinaceous and fatty compounds, thus, we determined element concentration (%) for C/H/N/S at the Department of Chemistry (University of Calgary), using the *Elementar UNICUBE* analyzer. An aliquot weighing 1g per starch taxon was combusted in a catalyst-loaded tube at 1150⁰ C. Then, the oxidized materials went through a Cu-loaded reduction tube (850⁰ C). The final products (N2, H2O, CO2 and SO2) were separated by a thermal conductivity detector (Table 1).

### FTICR-MS analysis

Extracts were analyzed with a 12T Bruker SolariX Fourier transform ion cyclotron resonance mass spectrometer. Each day, before analysis, an in-house standard was run to compare it to previous results and if there were inconsistencies, we carried out routine instrument calibration [60, 61]. Samples were analyzed through electrospray ionization in negative ion mode (ESI-N) to investigate species with deprotonation potential [62, 63]. (Initially, we carried out some analysis in Atmospheric Pressure Photoionization positive ion mode. Yet, ionization was better in ESI-N, as most species formed through the Maillard reaction contain de-protonatable hydroxyl and carboxyl functional groups.) Analyte flow rate was 200 μL / h, capillary voltage was 4 kV, and the nebulizer pressure set at 0.5 bar. Spectra were collected in absorption mode, using the algorithm developed by our colleagues [64]. Two hundred transients of 8,000,000 points were collected and summed to improve our experimental signal / noise ratio.

FTICR-MS raw data were processed using the calibration and peak assignment software CaPA v.2.2 (Aphorist Inc.). Peaks with signal / noise ratio > 5 were assigned on m / z measurements and stable isotopic pattern to the third most intense isotopologue. The elemental composition boundaries were set at ^12^C_4-95_, ^1^H_0-200_, ^16^O_0-30_, ^14^N_0-8_, ^32^S_0-2_, ^23^Na_0-2_, ^15^P_0-2_, and ^35^Cl_0-2_. Only species with mass errors < 300 p.p.b. were accepted. The mass spectra were internally calibrated using homologous peak series. Controls using solvent blanks identified contaminants to exclude. Identified peaks from the analysis of the Maillard reaction products were produced from single-charged ions, and < 10% of the total spectral ion intensity was left unassigned. Parent, monoisotopic peak height measured intensity. Ragnarök v.2.4 (Aphorist Inc.) was used for visualization and interpretation of multidimensional / multi-sample FTICR-MS data. Molecular masses and ring number / unsaturation levels for each chemical species were determined. We assessed double bond equivalents (DBE) as (CcHhNnSsOo = c-h/2+n/2+1). In addition, we derived lumped, summed abundances of whole heteroatom classes, double bond equivalents group distributions within a heteroatom class, carbon number distributions of consolidated samples including all ions, groups of compound classes and individual groups of pseudo-homologous compounds, and plots for related groups. For the visualization of MS data displaying hundreds of organic molecular formulae two-dimensionally, we utilized van Krevelen diagrams [65], Kendrick plots [60], as well as their modified versions.

## Results

### Characterization of Maillard reaction products based on atomic ratios (H/C, O/C, N/C, N/O) and compound class distribution

**Fig 1:**
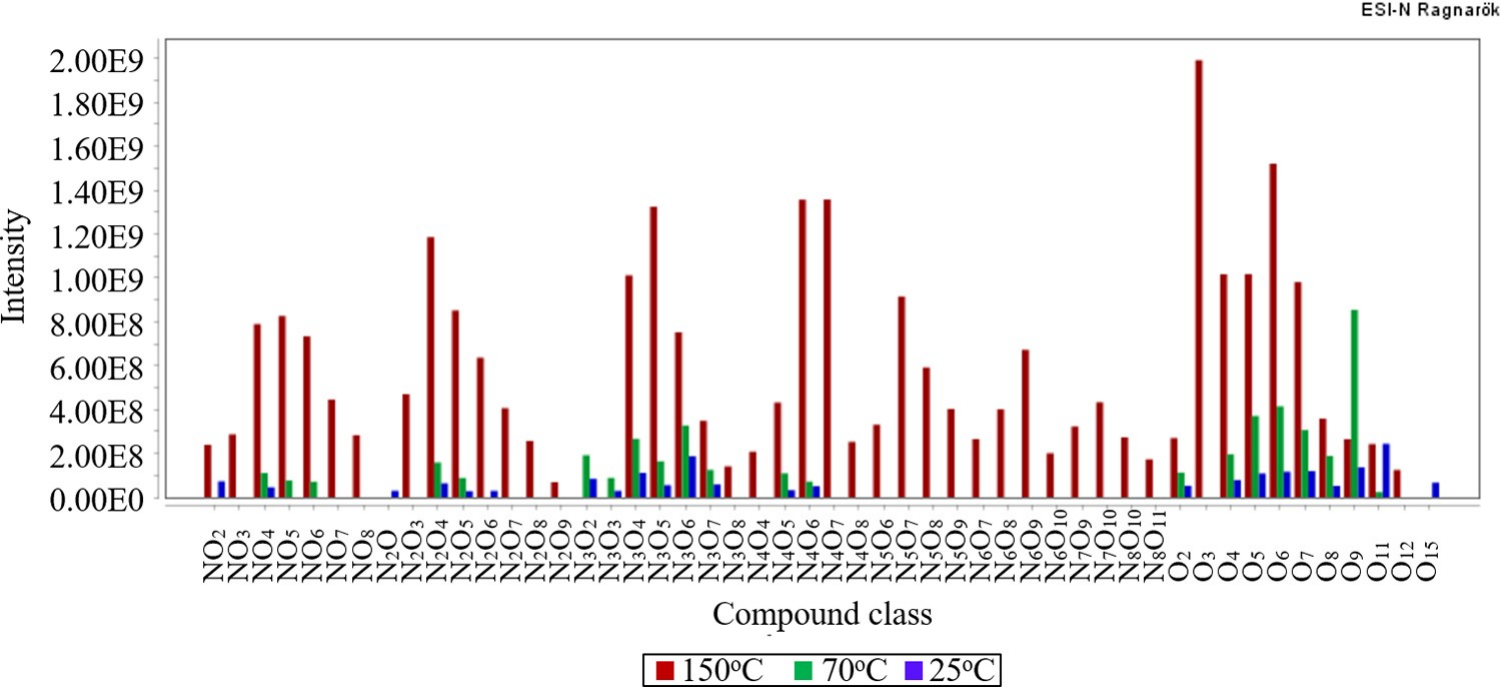
Relative abundance of heteroatom compound classes analysed with FTICR-MS, electrospray ionization in negative ion mode. Note the changes in MRP distribution after the reaction of glycine with *Ipomoea longituba* starch at 150°C, 70°C, and 25°C.

**Fig 2:**
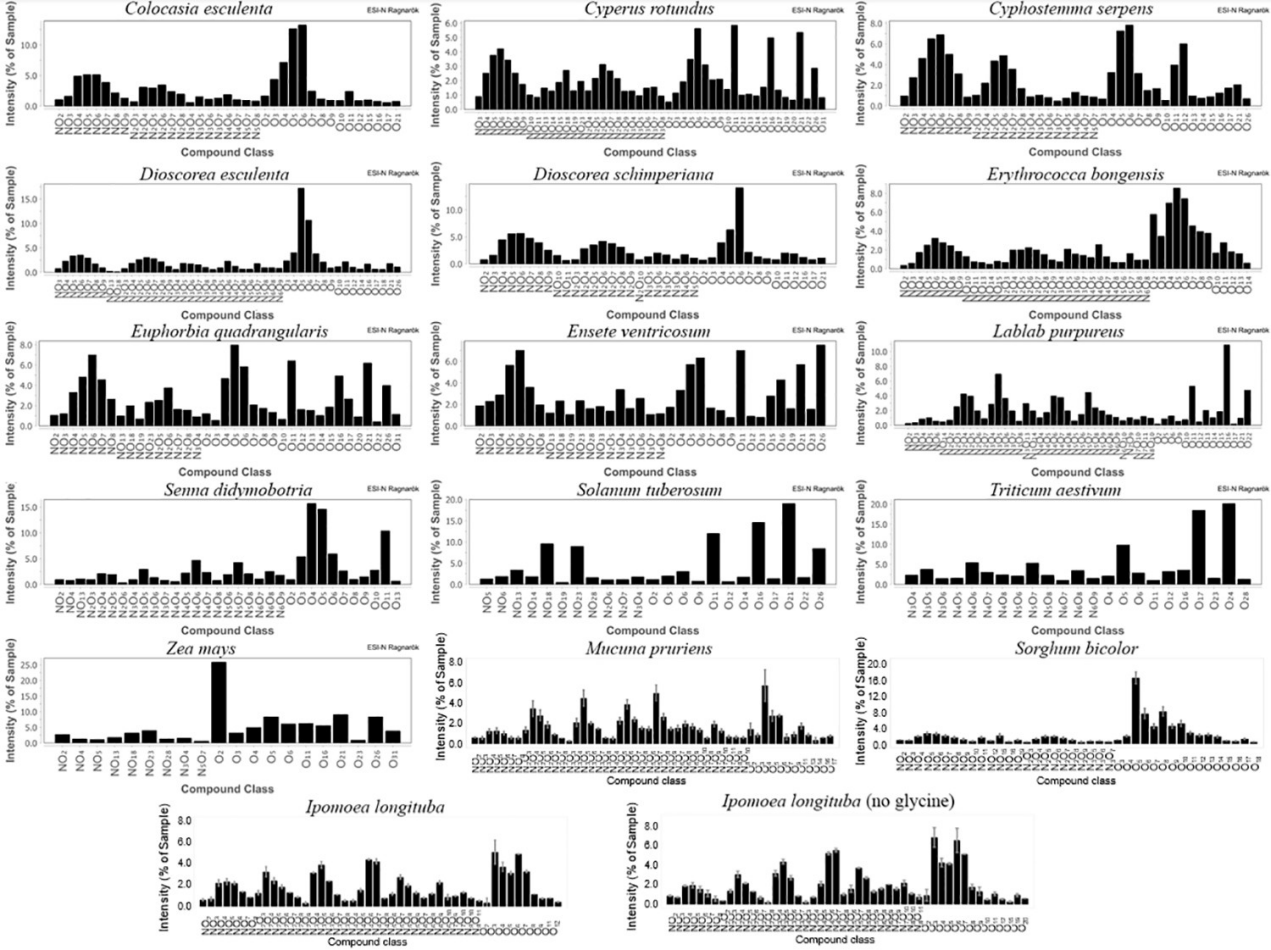
Compound class distribution of water-soluble reaction products (melanoidins). Reaction of glycine and starch from the taxa listed in Table 1 (150°C, 24 h). FTICR-MS through electrospray ionization in negative ion mode. Controls in triplicate, with deviation bars: *Mucuna pruriens*, *Sorghum bicolor*, and *Ipomoea longituba*.

**Fig 3:**
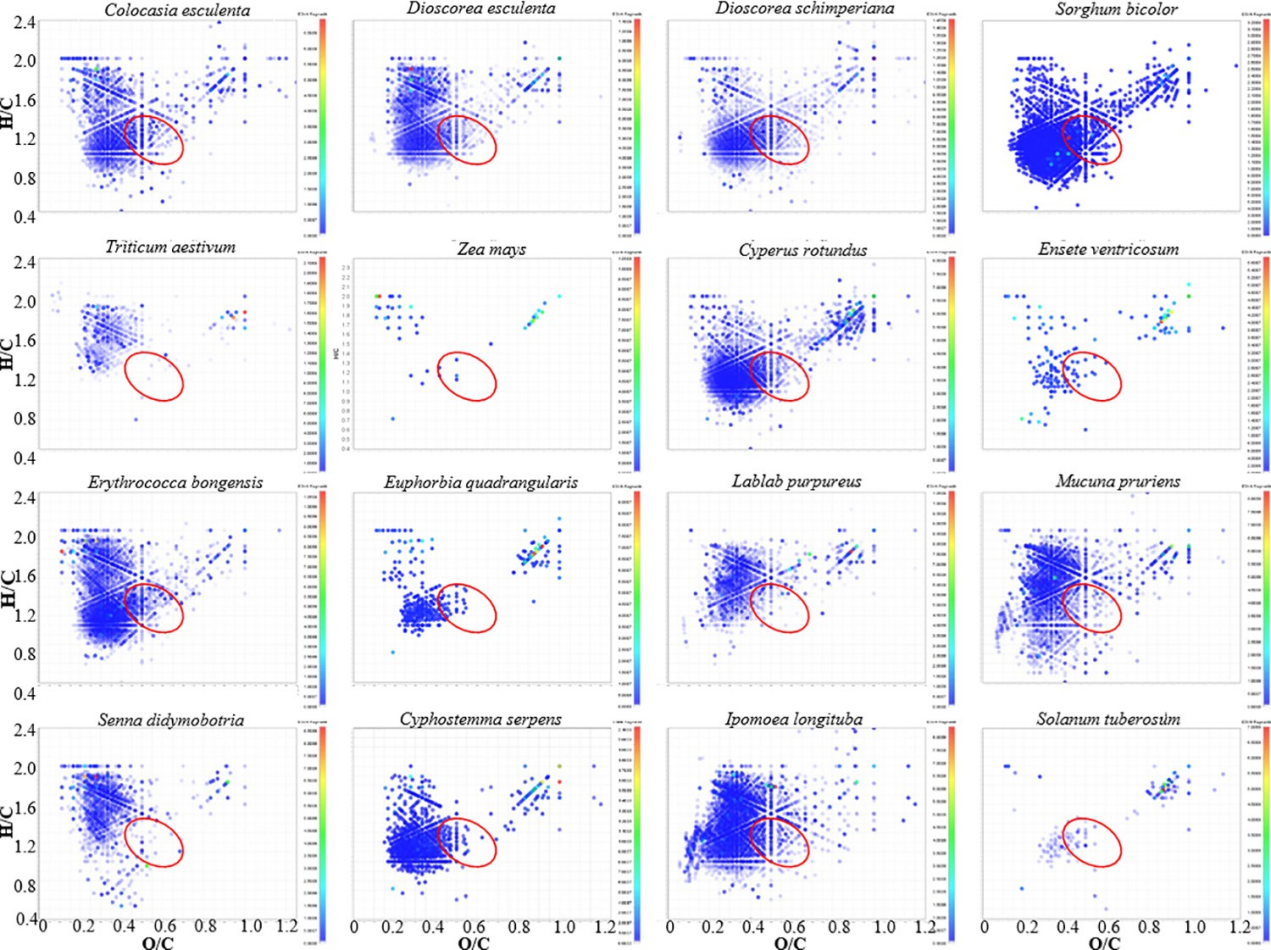
Van Krevelen diagrams illustrating the distribution of Maillard reaction products from hydrous pyrolysis of glycine and starch analysed through FTICR-MS electrospray ionization in negative ion mode. Each dot reflects at least one species. The red circle encompasses species within the ‘island of stability’ observed in refractory dissolved organic matter [66]. The red end of the scale indicates a higher relative abundance of species intensity.

In one of our model organisms, *Ipomoea longituba*, we show that temperature governs reactions leading to changes in the number and molecular distribution of heteroatom classes at 25°C, 70°C, and 150°C (24 h) (S1 Fig, Fig 1). For example, at 70°C there were minor variations relative to those in the glycine—starch at 25°C, with the only changes being the absolute abundance of some heteroatom classes and the many species containing exclusively oxygen heteroatoms. In contrast, at 150°C the number of resolvable heteroatom compound classes increased from 23 (70°C) to 46 (150°C), with the absolute number of peak counts, including isotopologues, surging from 944 (25°C) to 2,404 (70°C) and 5,307 (150°C) with total ion intensities of 4.8 E09, 1.4 E10, and 3.9 E10, respectively. At 150°C, the molecular weights of the MRPs reached 1,200 Da for individual heteroatom compounds. Double bond equivalents (1–22) demonstrated extensive cyclisation and unsaturation compared to those of glycine and glucose, with just one double bond equivalent each, per monomer. In short, the greater number and complexity of MRPs at higher temperatures (proxy for diagenesis), dictated a focus on reaction products at 150°C for the rest of the starch reference collection:

Major differences in the number and distribution of molecular compound classes appeared (Fig 2), ranging from 20 (*Zea mays*) to 50 (*Sorghum bicolor*, *Lablab purpureus*). MRP diversity is manifest in the Van Krevelen diagrams (Fig 3) that show fewer MRP species for some starches (*Ensete ventricosum*, *Euphorbia quadrangularis*, *Zea mays*, *Solanum tuberosum*), and more for others (*Colocasia esculenta*, *Cyperus rotundus*, *Cyphostemma serpens*, *Lablab purpureus*, *Senna didymobotria*, *Triticum aestivum*). The largest accumulation of reaction products occurred in *Dioscorea esculenta*, *Dioscorea schimperiana*, *Erythrococca bongensis*, *Ipomoea longituba*, *Mucuna pruriens*, and *Sorghum bicolor*.

**Fig 4:**
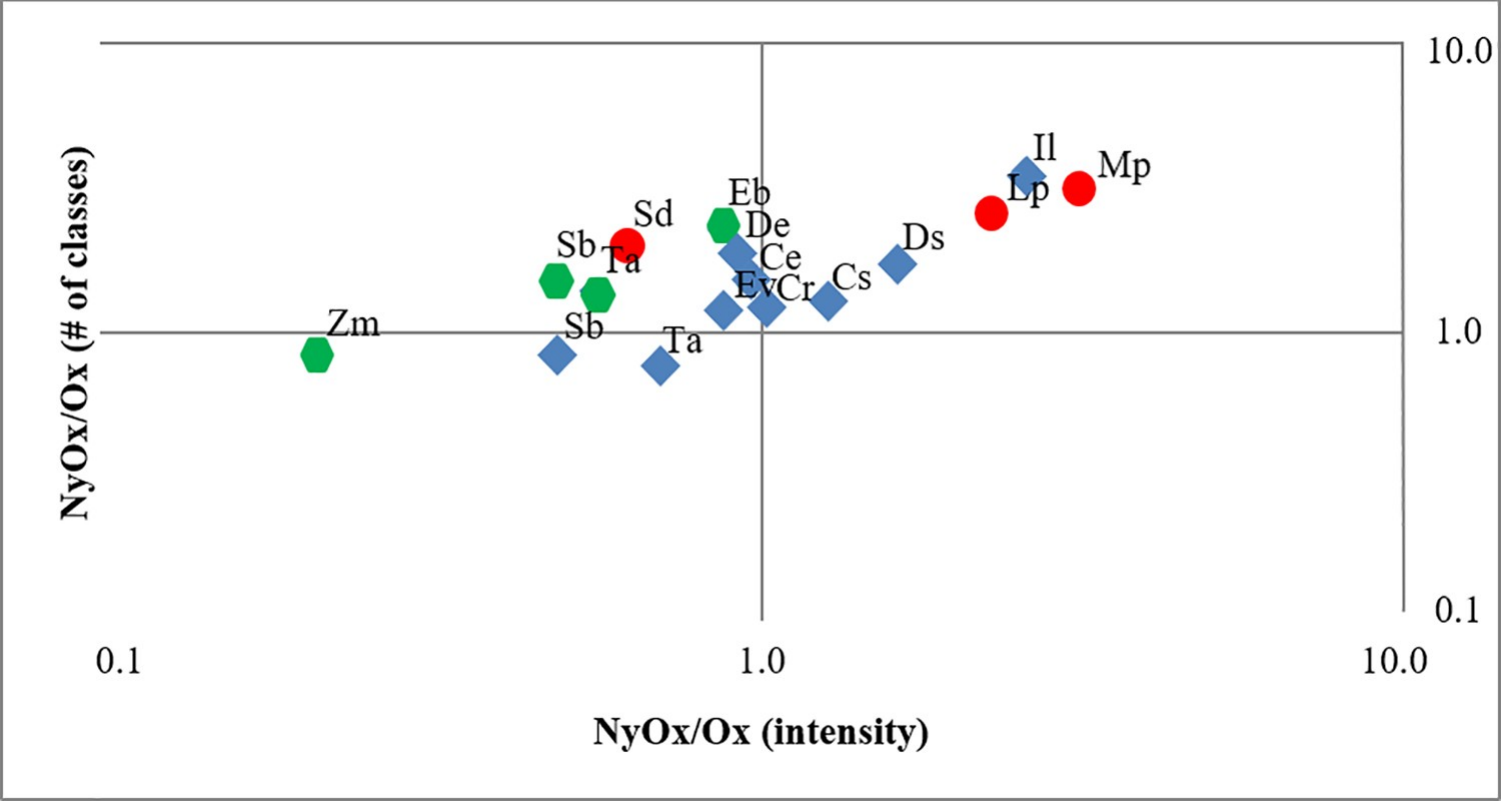
Compound class variation for nitrogen- and oxygen-containing NyOx, and oxygen-only Ox species. Relative intensity and number of compound classes from the Maillard reaction products generated by the reaction of starch mixed with glycine. FTICR-MS analysis, through electrospray ionization in negative ion mode. Initials are from the taxa listed in Table 1.

Fig 4 shows MRP species as a ratio of heteroatom group classes, summed intensity of constituents holding both nitrogen and oxygen atoms, and those with oxygen only in their molecular structure. Even though there is some correlation between the number of nitrogen-containing compound classes and the proportional intensity of these species, the differences in MRP mixtures are marked. The number of N_y_O_x_ classes is always greater than O_x_ classes in all MRPs, with only three exceptions (*Euphorbia quadrangularis*, *Zea mays*, *Solanum tuberosum)*. When weighted by intensity, oxygen-only heteroatom containing species show higher summed abundances in nine out of sixteen starches.

### Variations in molecular distribution

**Fig 5:**
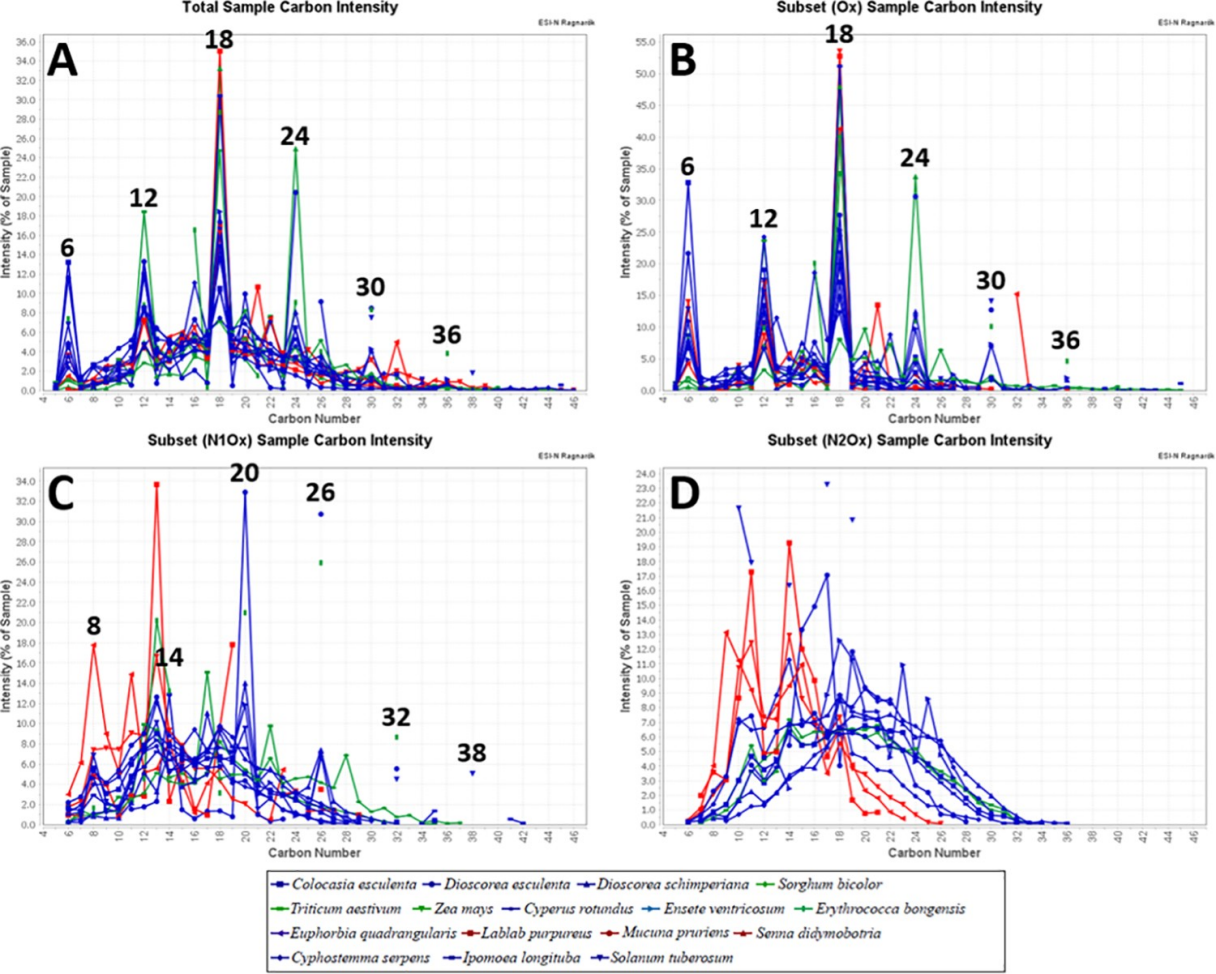
Consolidated carbon number distribution for all Maillard reaction products. A- Overall total carbon number distribution. B- Species containing only oxygen as heteroatom per molecule (Ox). C- Species containing one nitrogen and oxygen atoms (NOx). D- Species with two nitrogen and oxygen atoms (N2Ox). Most peaks reflect neo-formed, polysaccharide relics: A, B. Aditional two-carbon and one nitrogen may reflect simple polysaccharides plus single amino acid carbon skeletons (C). The predominance of species with incorporation of two glycine units into the newly formed molecules is less evident in (D).

Molecular size and, to some extent, structure are quantified by carbon number distributions in MRPs. Fig 5A shows overall carbon number distribution for all species, whereas species containing no nitrogen (i.e. only oxygen heteroatoms in molecule) are illustrated in Fig 5B. Species containing one nitrogen and oxygen heteroatoms per molecule (NO_x_) are in Fig 5C, and species with two nitrogen atoms and multiple oxygens as heteroatoms per molecule (N_2_O_x_) appear in Fig 5D. There is a clear dominance of species with carbon number multiples of six, along with increases in one double bond equivalents and five oxygens per unit (i.e. 6 C, 6 O, 1 DBE; 12 C, 11 O, 2 DBE; 18 C, 16 O, 3 DBE; 24 C, 21 O, 4 DBE). For oxygen-containing species, we see similar carbon number distributions, and copious O_x_ species indicating altered starch (carbohydrate) oligomers. Regarding nitrogen atoms (NO_x_), there is predominance of chemical species with two additional carbon atoms and one nitrogen atom, reflecting a combination of starch related moieties with one glycine backbone component. At higher nitrogen numbers (N_2_O_x_), the inordinate complexity of reactions and the diversity of products makes the linking of precursors and products unreliable.

**Fig 6:**
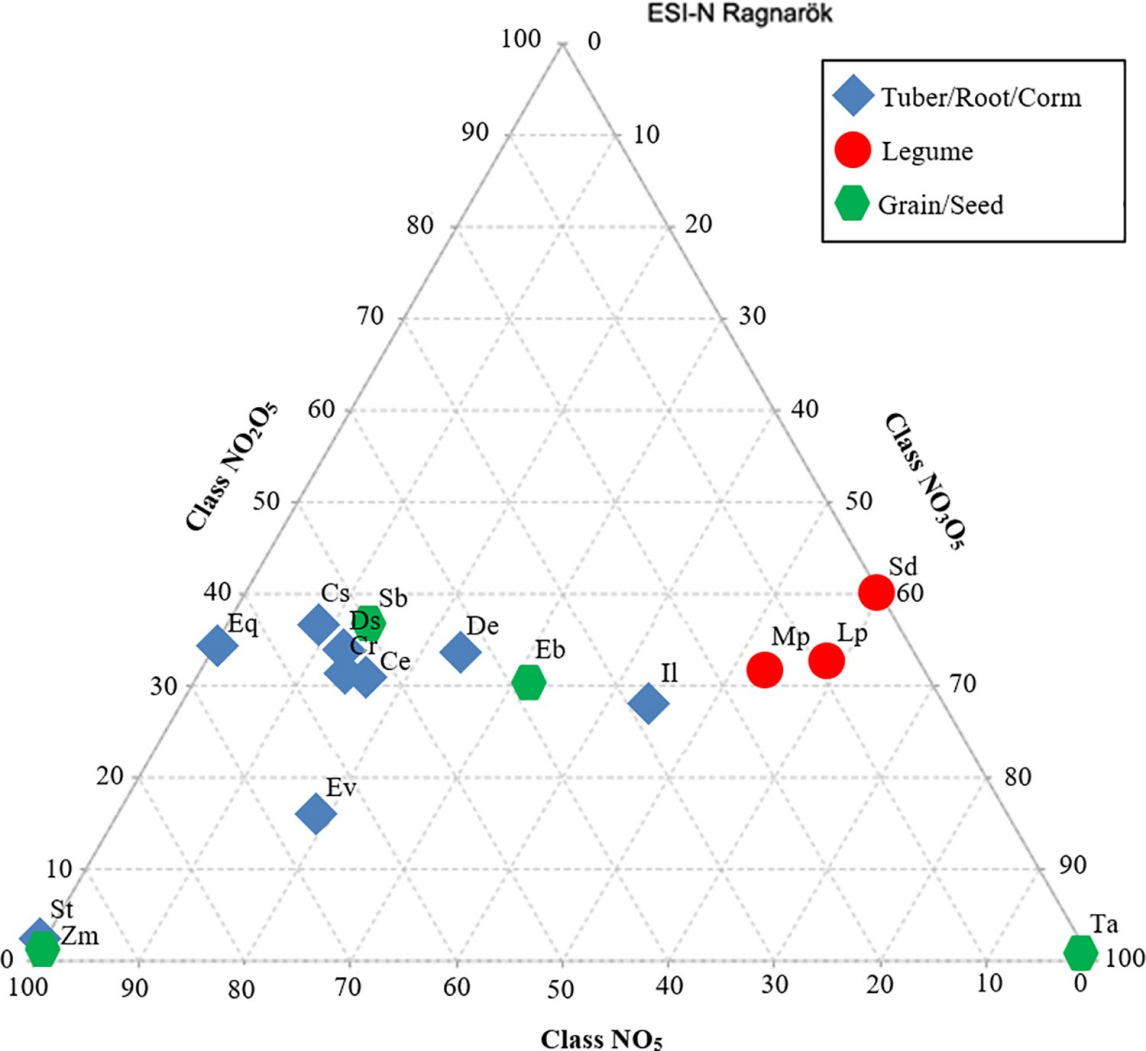
Triangular plot shows relative intensities of the classes NO_5_, N_2_O_5_ and N_3_O_5_ for Maillard reaction products from the reaction of starch with glycine. The initials represent the starch species listed in Table 1. The high proportion of N_3_O_5_ species denotes high reactivity (FTICR-MS, through electrospray ionization in negative ion mode).

The relative intensity distribution of compound classes (NO_5_ / N_2_O_5_ / N_3_O_5_) provides insights into the reactivity of the different starches (Fig 6). Overall, legume starches are the most reactive, with a maximum of N_3_O_5_ species, while the MRPs from tubers, roots, and corms are the least reactive and contain the greatest relative abundance of NO_5_ species, *Ipomoea longituba* aside. The MRPs from grains and seeds (*Erythrococca bongensis*, *Sorghum bicolor*) exhibit mid-range reactivity or plot as outliers due to lack of one or more of the studied compound classes (*Zea mays*, *Triticum aestivum*).

## Discussion

### The Maillard reaction products

Others have reported that compounds with up to four nitrogen atoms were identified in the Maillard reaction products of degraded carbohydrates with amino acids after heating at temperatures 150°C– 200°C and, in the case of reactions with cysteine, the heteroatom mixtures included some sulfur atoms [50]. By overall intensity, oxygen-only heteroatom species dominates, as observed by our study as well. In addition, they found caramelization products, including dehydration products of oligomers with loss of up to seven water molecules, oligomers of carbohydrates with up to six units, hydration products of sucrose oligomers, disproportionation products, and some aromatic species. Non-caramelization products (adducts of sucrose) were also detected. The similarity of Maillard reaction products from sucrose / amino acids [50] and starch / amino acids (this study) shows that these carbohydrates produce comparable MRPs.

Another study that provides relevant comparative information looked at the chemical changes occurring in the reaction of glycine with ribose [52]. The formation of reactive intermediates from Amadori rearrangement products and carbohydrate and amino acid degradation led to an exponential increase in the production of Maillard products at 100°C (2, 4, 6, and 10 h). Even though the majority of reaction products identified were under 400 Da, a large number of high molecular weight species appeared, demonstrating that degradation and condensation contribute to the diversity and molecular weight of Maillard reactions species.

The lower temperatures and heating times utilized by colleagues [52], compared to those in earlier work [50], show that the progression of the Maillard reaction is a function of time. Starting with glycine-ribose condensation, an extended series of dehydration reactions led to Maillard reaction products with more unsaturation and aromaticity [52]. The dehydration reactions were favored when the carbohydrate was covalently bond to the amino acid. These authors [52] concluded that oxygen-only reaction products have a different reactive behavior compared to species containing nitrogen and oxygen heteroatoms, and they linked 98% of the observed reaction products to seven types of transformations with the following mass differences: 2.01565 (2H); 12.00000 (Strecker degradation [+H_2_O-CH_2_O]); 18.01057 (H_2_O); 43.98983 (CO_2_); 57.02146 (glycine condensation); 75.03203 (glycine addition); 150.05283 (pentose addition).

Another work [53] used four amino acids in their Maillard reaction products. A pathway involving 73 products was common to all, with differences emanating from the nature of the amino acid side chain. The order of reactivity was identified as lysine > cysteine > isoleucine ~ glycine. The authors also compared the reaction products of six sugars (three pentoses and three hexoses) with glycine for 24 h at 100°C. They found that the number of Maillard reaction species varied with the sugar type. Pentoses produced more species than hexoses, with galactose and glucose producing the lowest numbers of products. However, as the characteristic positions of species in the van Krevelen diagrams did not change, they concluded that the amino acid precursor is primarily responsible for the individual characteristics of the detected Maillard reaction products, but did not influence the overall net reaction.

### Preservation pathway

One pathway to organic matter preservation that has received little attention in archaeological sciences is the formation of humic and kerogeneous materials. These have high molecular weights and are less accessible to microorganisms [67, 68]. The Maillard reaction produces complex mixtures from simple biochemical precursors (S2 Fig). During this browning reaction, glucose (starch) and amino acids produce glycosylamine, forming an Amadori rearrangement product known as a ketose. The subsequent degradation of this component is pH-dependent. At pH < 7, it undergoes 1, 2 -enolisation with the formation of furfural or hydroxymethylfurfural (C_6_H_6_O_3_). At pH > 7, the degradation of Amadori compounds involves 2, 3- enolization, in which reductones such as 4-hydroxy-5-methyl-3-furanone (C_5_H_6_O_3_), as well as acetol (C_3_H_6_O_2_), pyruvaldehyde (C_3_H_4_O_2_), and diacetyl (CH_3_CO)_2_ are created. Later, cyclizations, dehydrations, retroaldolizations, rearrangements, isomerizations, and further condensations occur, leading to complex brown nitrogenous polymers and co-polymers.

All the MRPs show the same basic distribution of heteroatom groups and / or double bond equivalent distributions, yet differences among starch taxa and plant parts do emerge. Although Amadori rearrangement products are the main Maillard reaction pathway, recent advances in ultrahigh resolution mass spectrometry have allowed for a more comprehensive, non-targeted analysis of reactive intermediates as well. Golon et al. [50] investigated the reaction products of mixtures of sucrose and amino acids at temperatures 150°C—200°C. Several thousands of reaction products were identified with oxygen-only heteroatom species dominating by overall intensities over products with one nitrogen and oxygen heteroatoms. The latter class contained the largest number of compounds. We made similar observations in our experiments. We studied if individual starch taxa transfer distinctive information to their respective MRPs. All but three sets from our starch-glycine mixtures contain more N_y_O_x_ than O_x_ classes, whereas nine out of sixteen showed higher overall intensities of oxygen-only species compared to the mixed nitrogen-oxygen species. With great proportions of nitrogen functional groups in the studied Maillard reaction products, we suggest that many of these species will be adsorptive to mineral or artefact surfaces [22], further facilitating their preservation.

### Diagenetic recalcitrance of Maillard reaction products

Different biochemical precursors have variable resistance to alteration: lipids, lignins, and proteins are recalcitrant [59, 69], but carbohydrates rapidly decompose [70, 71]. Labile molecules do not retain chemical functionality [33, 72]. Furthermore, in palaeosciences, well-preserved physical structure does not imply chemical conservation [58, 70]. Starch-derived reaction products have potential for diagenetic resistance, as suggested by bulk compositions combining both relict and neo-formed species and prior work indicating similarities between MRPs and stable carbon forms such as kerogens [49]. Moreover, there are systematic differences in MRPs among starch taxa as per botanical provenance. The relative intensity distribution of heteroatom classes NO_5_, N_2_O_5_ and N_3_O_5_ in MRP suggests that the starch from different plant parts is variably reactive, with exceptions. Legume starch is very reactive as shown by higher proportions of N_3_O_5_ species, with grain starch being intermediate, whereas tuber starch has lower reactivity. In addition, our experiments on starch degradation without glycine demonstrate that the complex chemistry of native starch is, in itself, capable of producing Maillard reaction products. Significantly, the three starches that were industrially isolated or purified (*Zea mays*, *Triticum aestivum*, and *Solanum tuberosum*) are the ones harboring the lowest relative intensities of nitrogen-containing species (Fig 4, cf. Fig 6).

While some chemical species will degrade beyond recovery, others, especially condensed, cyclic, and potentially aromatic species, will be more resistant to biodegradation. We note that recalcitrant molecular species, comprising biotic (microbial degradation) and abiotic (photo-oxidation) reaction processes, transform organic matter from terrestrial and marine environments into a fraction called dissolved organic matter (DOM), that ultimately accumulates in oceans with turnover times in excess of 24,000 years [66, 73]. This long-lived pool of organic carbon has also been suggested as a reservoir for analogous synthetic carbon storage vectors produced from biomass as part of a large-scale carbon dioxide removal process for climate mitigation [74]. While controversial, molecular comparisons of model Maillard reaction products using FTMS with DOM species in marine waters of age like many archaeological contexts, suggest such Maillard reaction products may contribute, in part, to some of the stable organic species in marine DOM [75, 76]. Although speculative, this suggests that even soluble Maillard products may have some degradation resistance.

**Fig 7:**
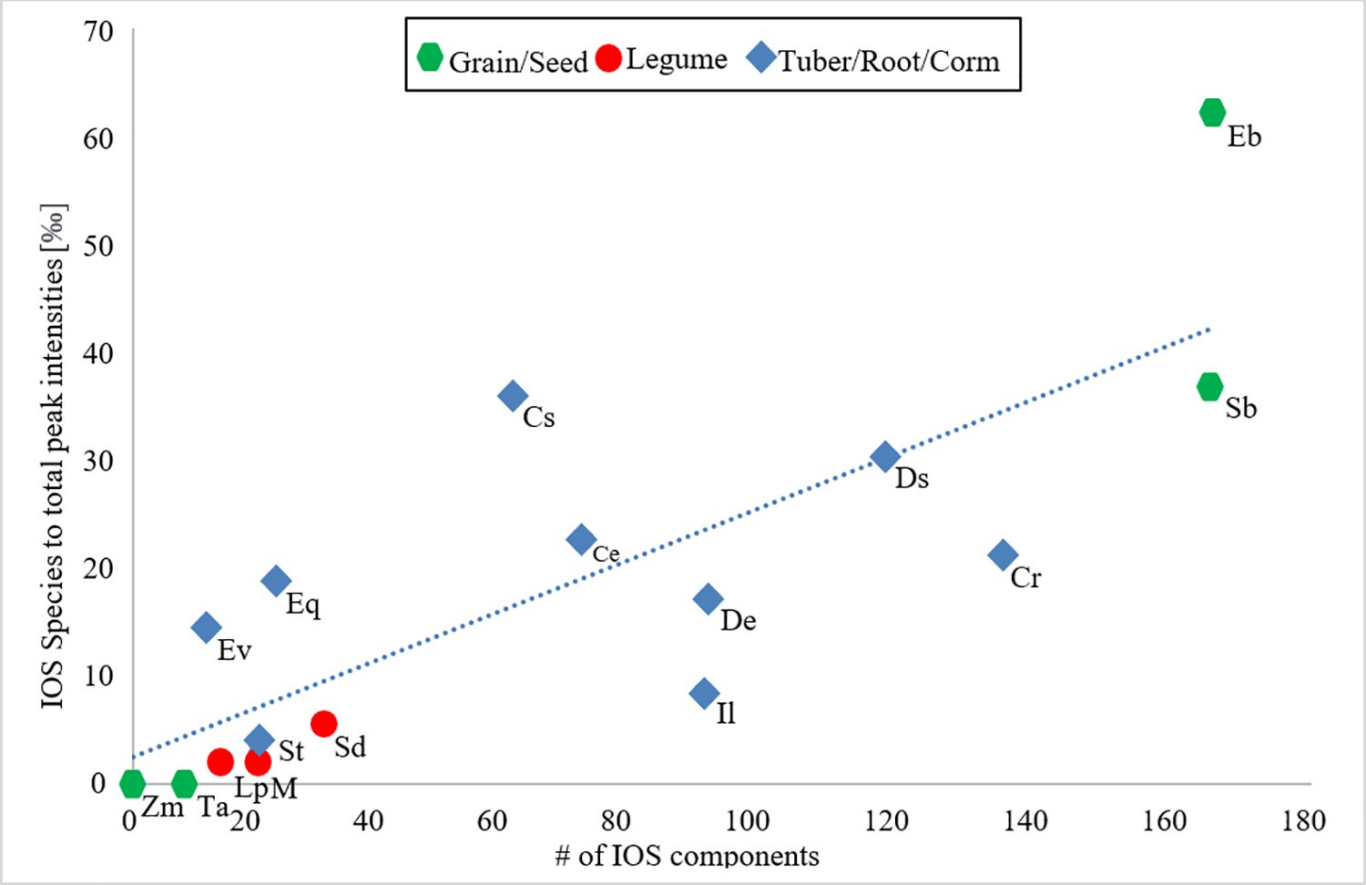
Total number of ‘Island of Stability’ (IOS) species compared to their relative total peak intensity. Number of IOS components estimates the amount of recalcitrant Maillard reaction products formed in starch–glycine experiments.

**Fig 8:**
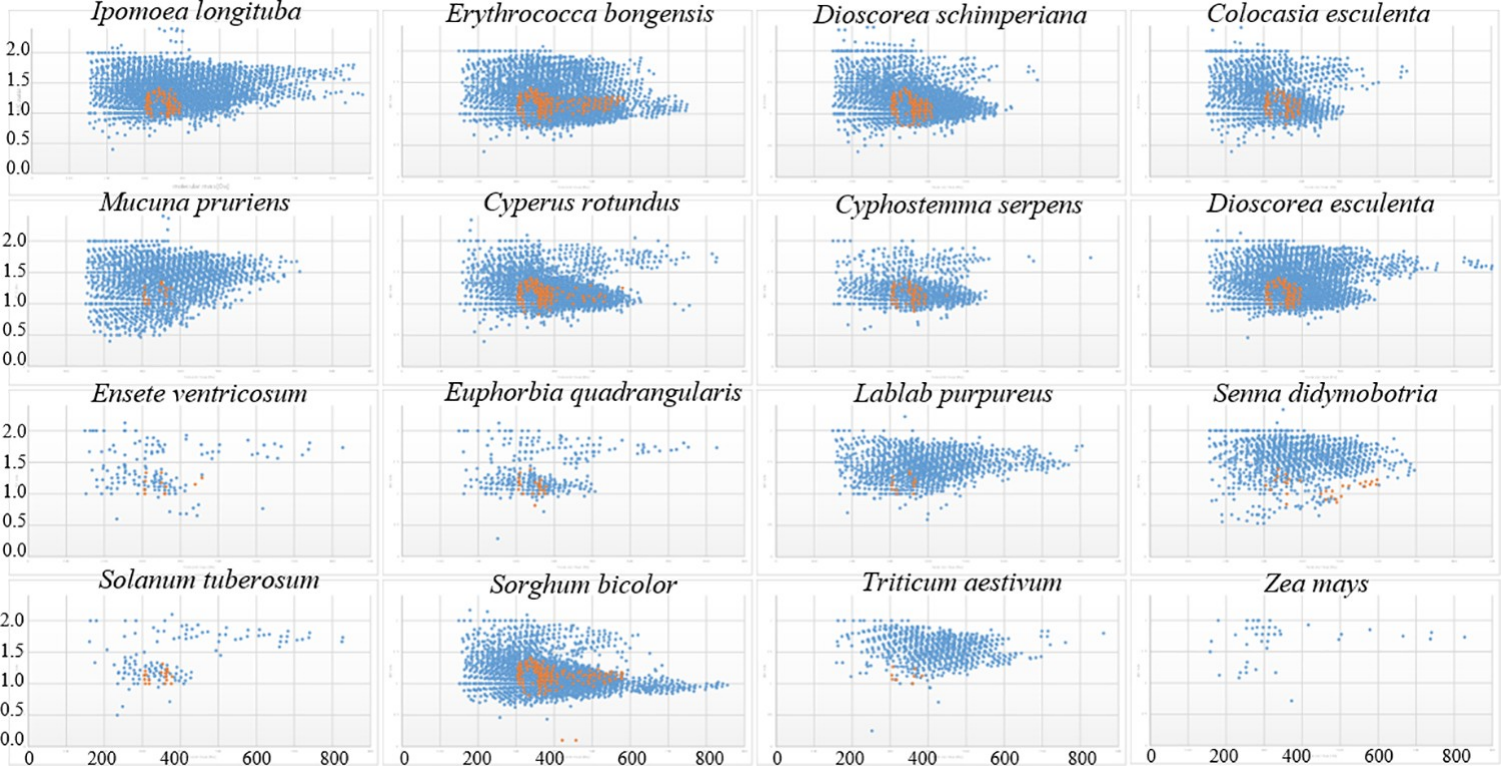
Compositional space for total assigned peaks (blue) and ‘island of stability, IOS’ species (orange), shown as H / C ratio versus molecular mass. IOS species defined as H / C ratio (1.04–1.3), molecular mass: 300–600 Da.

The analysis of oceanic organic matter from a molecular dataset [66] shows chemical species with a stable molecular composition within an atomic ratio of H / C (1.17 ± 0.13) | O / C (0.52 ± 0.10) and molecular masses of 360 ± 28 and 497 ± 51 Da. This compositional space is called the ‘island of stability’ (IOS). Even though the feedstock for marine organic matter is different, it still is a mixture of polysaccharide and proteins in both settings. Thus, we looked for counterpart species in our starch experiment to those within the island of stability as potential markers of stable species in deep time. Most IOS species in our study are in the 300 Da—400 Da molecular range (Fig 7). The condensed and recalcitrant character of these species (Table 2) is manifest in their lower carbon numbers (17.4 ± 1.9) and higher double bond equivalents (9 ± 1.4), compared to those in total Maillard reaction products: 18.5 ± 6 and 7.5 ± 3.4, respectively. The greatest diversity and frequency of recalcitrant species (Fig 8, Table 2) were found in two grains: *Erythrococca bongensis* (Eb) and *Sorghum bicolor* (Sb). The largest and one of the richest clusters occurs among all the underground storage organs, with the exception of potato. The corm and root starch from *Ensete ventricosum* (Ev) and *Euphorbia quadrangularis* (Eq) contain higher relative intensities of recalcitrant species than the two grains mentioned above and all legumes.

**Table 2:** Chemical summary of recalcitrant species with potential for deep time preservation.

*Taxon*	MI* Peak Count		*Average Mass*		*Average Carbon #*		*Average DBE* *	
	*Total*	IOS*	Total	IOS*	Total	IOS*	Total	IOS*
*Ipomoea longituba*	2910	87	404.3 ± 133.9	348.4 ± 27.7	19.7 ± 7.1	16.9 ± 1.6	8.6 ± 3.7	8.5 ± 1.3
*Cyperus rotundus*	2195	133	393.8 ± 106.8	372.5 ± 54.6	19.7 ± 5.7	18.0 ± 2.8	9.1 ± 4.0	8.9 ± 1.6
*Sorghum bicolor*	2277	164	394.9 ± 122.4	394.7 ± 73.0	20.2 ± 6.9	18.9 ± 3.5	10.3 ± 4.6	9.0 ± 1.9
*Ensete ventricosum*	148	11	350.2 ± 148.1	355.4 ±49.1	16.2 ± 5.5	17.3 ± 1.9	6.4 ± 3.8	8.7 ± 1.2
*Lablab purpureus*	1266	13	399.7 ± 120.3	337.1 ± 25.6	18.4 ± 5.8	16.2 ± 0.9	7.8 ± 3.0	8.7 ± 0.9
*Triticum aestivum*	784	8	429.5 ± 139.2	334.8 ± 31.0	20.0 ± 8.0	16.3 ± 1.3	7.2 ± 3.0	9.5 ± 1.9
*Dioscorea esculenta*	2375	88	393.3 ± 109.2	353.3 ± 26.2	19.6 ± 5.8	17.1 ± 1.6	8.0 ± 3.4	8.6 ± 1.3
*Zea mays*	44	4	367.9 ± 197.3	231.1 ± 33.5	17.3 ± 6.2	11.0 ± 2.0	3.9 ± 2.7	5.8 ± 0.5
*Solanum tuberosum*	136	19	392.1 ± 154.0	342.2 ± 26.4	16.8 ± 5.9	16.5 ± 1.4	6.1 ± 3.1	9.0 ± 0.8
*Cyphostemma serpens*	945	58	362.9 ± 89.1	350.1 ± 29.9	18.6 ± 4.9	17.5 ± 1.7	8.8 ± 3.3	8.9 ± 1.3
*Dioscorea schimperiana*	2104	115	356.6 ± 97.7	355.6 ± 30.4	17.5 ± 5.6	17.0 ± 1.6	8.5 ± 3.9	8.9 ± 1.4
*Euphorbia quadrangularis*	327	22	361.5 ± 112.8	343.1 ± 26.3	17.6 ± 4.9	17.2 ± 1.6	7.3 ± 3.4	8.9 ± 1.3
*Senna didymobotrya*	1106	29	393.4 ± 108.7	461.8 ± 93.9	19.1 ± 5.3	21.1 ± 4.0	6.6 ± 3.1	10.7 ± 2.2
*Erythrococca bongensis*	3061	165	402.7 ± 108.3	401.8 ± 76.7	20.3 ± 6.0	19.4 ± 3.7	9.1 ± 4.1	9.4 ± 1.7
*Colocasia esculenta*	1394	69	323.9 ± 88.4	350.5 ± 29.5	15.8 ± 4.7	17.0 ± 1.5	7.1 ± 3.5	8.9 ± 1.3
*Mucuna pruriens*	1872	19	369.4 ± 113.2	340.8 ± 26.1	17.7 ± 6.3	16.9 ± 1.7	8.0 ± 3.6	8.7 ± 1.0
***Grand Average***	**1375**	**55**	**384.4 ± 124.3**	**360.1 ± 39.6**	**18.5 ± 6.0**	**17.4 ± 1.9**	**7.5 ± 3.4**	**9.0 ± 1.4**

* MI = Monoisotopic* IOS = Island Of Stability

** DBE = Double Bond Equivalents.

## Conclusions

We put forward a novel geochemical method to fingerprint decayed starch microstructurally. The Maillard reaction produces thousands of identifiable compounds in a simple system that has a counterpart in both culinary manipulation and archaeological site formation processes, bringing together starch and amino acids through processing, handling, and sedimentation. Some of the diagenetic products encountered are rich functional groups prone to adsorption, refractory, and could reasonably be expected to occur in archaeological residues and / or mineral substrates associated with artifacts or encasing them. These alteration products are proxies of starch precursors, facilitating an alternative route to study ancient starch that has the potential to overcome the taphonomic, diagenetic, and contamination limitations inherent to conventional archaeological methodologies that rely on native starch granules.

High resolution mass spectrometry can characterize starchy residue, with neo-formed compounds including an extensive variety of molecular weights and chemistries. Despite all Maillard reaction products showing the same heteroatom groups and / or double bond equivalent distributions, differences among starches do arise, with starch from underground storage organs standing the best chance for long-term archaeological preservation and taxonomic identification. Thus, FTICR-MS provides a molecular chemotaxonomy. Developing an extraction protocol for these alteration products and identifying starch residues from prehistoric artifacts are the next logical step. While this is a major task yet to be undertaken, our studies suggest that the alteration products will have high molecular weights, will contain many heteroatoms, and will have low solubility in traditional solvents. Fortunately, new solvent classes have appeared in the last few decades, and ionic liquids have a wide range of customizable properties [77], suggesting this will be one route to explore as we seek a viable extraction protocol. Other routes will link imaging and advanced mass spectrometry systems, such as MALDI-FTMS approaches and derivatives [78]. While various methods will undoubtedly ensue, there is no current substitute for skilled archaeological insights to select appropriate settings and samples for biogeochemical and materials science examination.

## Supporting information

S1 Fig Van Krevelen (H/C vs O/C) and modified van Krevelen (N/C vs N/O) diagrams illustrating Maillard reaction products from hydrous pyrolysis of glycine and *Ipomoea longituba* starch (150°C, 24 h) analyzed with FTICR-MS in electrospray ionization in negative ion mode. Each dot reflects at least one melanoidin species formed. Green star symbol indicates the elemental composition of glycine, and the red star symbol plots the starch monomer. Relative abundance of species intensity increases towards the end of the red scale.
(DOCX)

S2 Fig Chemical summary of the Maillard reaction after Martins et al [79], modified from Hodge [48]. (DOCX)
