# Supplementary material for: A novel route for identifying starch diagenetic products in the archaeological record
Source: PLoS One. 2021 Nov 18;16(11):e0258779. doi: 10.1371/journal.pone.0258779 (PMC8601532; doi:10.1371/journal.pone.0258779)
Supplement: S1 Fig — Each dot reflects at least one melanoidin species formed. Green star symbol indicates the elemental composition of glycine, and the red star symbol plots the starch monomer. Relative abundance of species intensity increases towards the end of the red scale. (DOCX) [file pone.0258779.s001.docx]

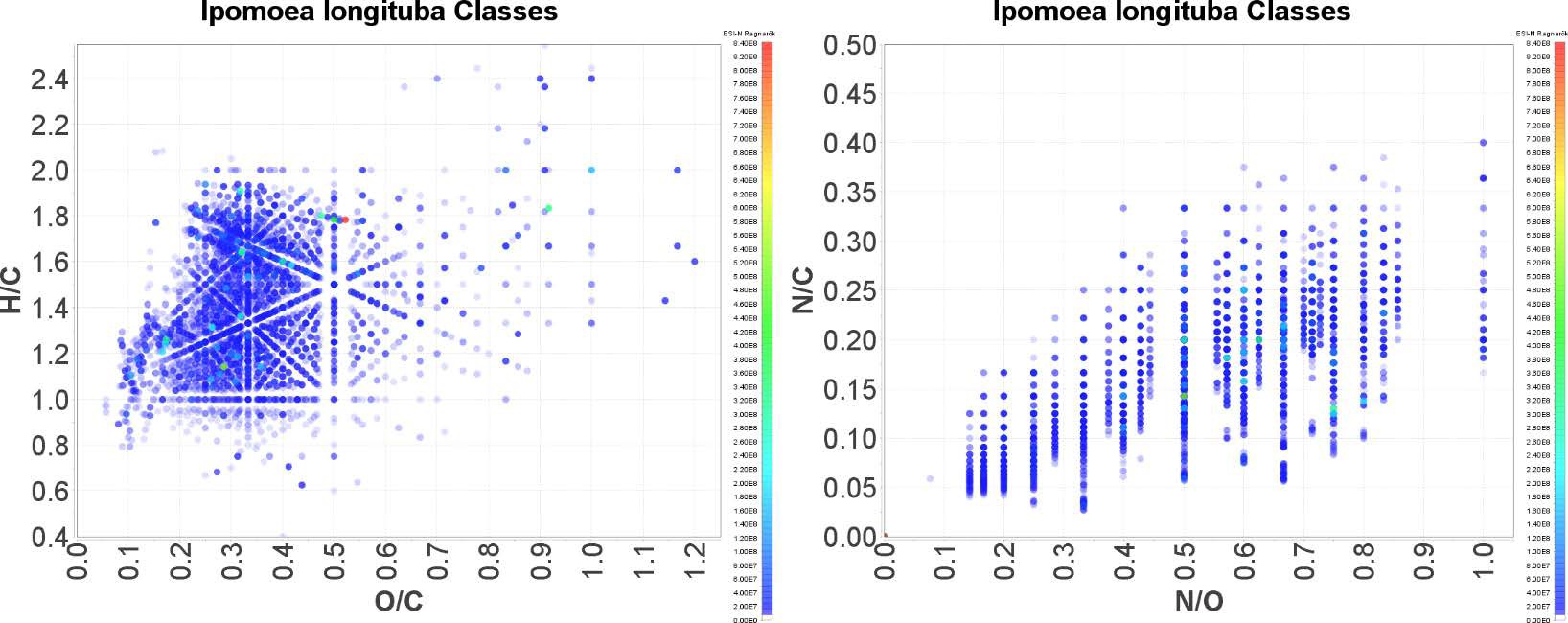

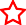

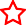

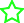

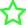


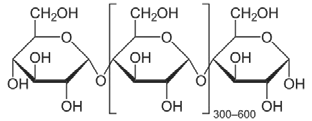


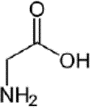

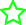
 Glycine

H/C = 2.5 N/O = 0.5

O/C = 1 N/C = 0.5


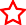
 Starch

H/C = 1.66 N/O = 0

O/C = 0.84 N/C = 0

**S1 Fig. Van Krevelen (H/C vs O/C) and modified Van Krevelen (N/C vs N/O) diagrams illustrating Maillard reaction products from hydrous pyrolysis of glycine and *Ipomoea longituba* starch (150 °C, 24 h) analyzed with FTICR-MS in electrospray ionization in negative ion mode. Each dot reflects at least one melanoidin species formed. Green star symbol indicates the elemental composition of glycine, and the red star symbol plots the starch monomer. Relative abundance of species intensity increases towards the end of the red scale.**
