# Supplementary figures and images for: A novel route for identifying starch diagenetic products in the archaeological record
Source: PLoS One. 2021 Nov 18;16(11):e0258779. doi: 10.1371/journal.pone.0258779 (PMC8601532; doi:10.1371/journal.pone.0258779)

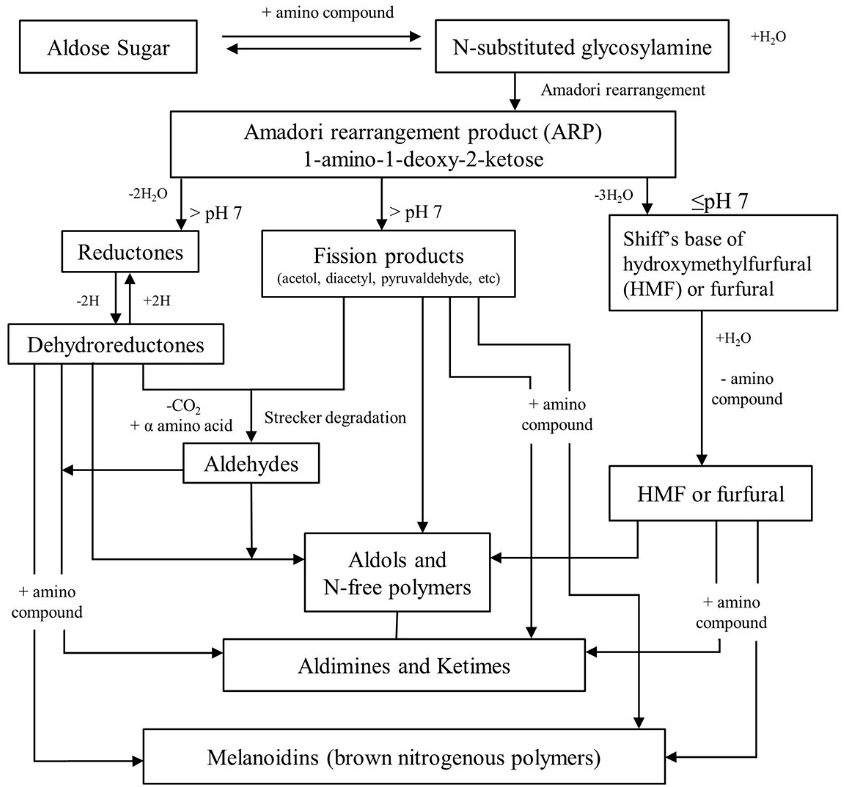


**S2 Fig. Chemical summary of the Maillard reaction after Martins et al [79], modified from Hodge [48].**

Supplement: S2 Fig — (DOCX) [file pone.0258779.s002.docx]
